# Water Management-Mediated Changes in the Rhizosphere and Bulk Soil Microbial Communities Alter Their Utilization of Urea-Derived Carbon

**DOI:** 10.3390/microorganisms12091829

**Published:** 2024-09-04

**Authors:** Peng Chen, Yawei Li, Yuping Lv, Junzeng Xu, Zhongxue Zhang, Xiaoyin Liu, Yajun Luan, Qi Wei, Ennan Zheng, Kechun Wang

**Affiliations:** 1 The National Key Laboratory of Water Disaster Prevention, Hohai University, Nanjing 210098, China; chenpeng_isotope@163.com (P.C.); yaweizx@hhu.edu.cn (Y.L.); xjz481@hhu.edu.cn (J.X.); 2 College of Hydrology and Water Resources, Hohai University, Nanjing 210098, China; 3 College of Agricultural Science and Engineering, Hohai University, Nanjing 211100, China; luan450705@163.com (Y.L.); weiqi0828@hhu.edu.cn (Q.W.); kechunw101@hhu.edu.cn (K.W.); 4 Jiangsu Province Engineering Research Center for Agricultural Soil-Water Efficient Utilization, Carbon Sequestration and Emission Reduction, Hohai University, Nanjing 211100, China; 5 College of Hydraulic Science and Engineering, Yangzhou University, Yangzhou 225009, China; lvyuping@yzu.edu.cn; 6 School of Water Conservancy and Civil Engineering, Northeast Agricultural University, Harbin 150030, China; zhangzhongxue@163.com; 7 School of Water Conservancy and Electric Power, Heilongjiang University, Harbin 150080, China; 2020024@hlju.edu.cn

**Keywords:** water-saving irrigation, ^13^C-urea, microbial community structure, ^13^C-PLFA

## Abstract

As one of the most important fertilizers in agriculture, the fate of urea-derived nitrogen (urea-N) in agricultural ecosystems has been well documented. However, little is known about the function of urea-derived carbon (urea-C) in soil ecosystems, especially which soil microorganisms benefit most from the supply of urea-C and whether the utilization of urea-C by the rhizosphere and bulk soil microorganisms is affected by irrigation regimes. To address this, a soil pot experiment was conducted using ^13^C-labeled urea to investigate changes in the composition of the rhizosphere and bulk soil microbial communities and differences in the incorporation of urea-derived C into the rhizosphere and bulk soil phospholipid fatty acids (PLFA) pool under flooded irrigation (FI) and water-saving irrigation (CI). Our results suggest that the size and structure of the rhizosphere and bulk soil microbial communities were strongly influenced by the irrigation regime. The CI treatment significantly increased the total amount of PLFA in both the rhizosphere and bulk soil compared to the FI treatment, but it only significantly affected the abundance of Gram-positive bacteria (G+) in the bulk soil. In contrast, shifts in the microbial community structure induced by irrigation regimes were more pronounced in the rhizosphere soil than in the bulk soil. Compared to the FI treatment, the CI treatment significantly increased the relative abundances of the G+ and Actinobacteria in the rhizosphere soil (*p* < 0.05). According to the PLFA-SIP, most of the labeled urea-derived C was incorporated into 16:1ω7c, 16:0 and 18:1ω7c under both treatments. Despite these general trends, the pattern of ^13^C incorporation into the PLFA pool differed between the treatments. The factor loadings of individual PLFAs suggested that 18:1ω7c, 16:1ω7c and 16:1ω5c were relatively enriched in urea-C in the bulk soil, while 17:1ω8c, i16:0 and 16:0 were relatively enriched in urea-C in the rhizosphere soil under different irrigation regimes. The loadings also confirmed that 10-me16:0, cy17:0 and cy19:0 were relatively enriched in urea-C under the CI treatment, whereas 14:0, a15:0 and 15:0 were relatively enriched in urea-C under the FI treatment. These results are helpful not only in revealing the interception mechanism of urea-C in soil but also in understanding the functions of key microbes in element cycles.

## 1. Introduction

Urea, as the fertilizer with the highest nitrogen content, is one of the most important nitrogen inputs in agriculture around the world [1]. More than half of the global population relies on this fertilizer for agricultural production [2], accounting for 73.4% of the global N application [3]. Therefore, it plays a crucial role in ensuring global food security. The transformation and fate of urea-N in plant–soil–microbe systems have been well documented to understand its role in soil N cycling and improving crop nitrogen use efficiency [4,5,6,7,8]. Carbon is the second most abundant element in urea and is an essential biogenic element for soil microorganisms. However, our understanding of the partitioning of urea-C among microbial groups in cropland soil is still limited.

After urea is applied to the soil, oxidizable N and HCO_3_^−^ are produced by the enzymatic hydrolysis of urea by urease. These products can be utilized as a source of C and N by nitrifying microorganisms (*Nitrosomonas europaea* and *Nitrosospira* sp.) [9]. Additionally, HCO_3_^−^ derived from urea hydrolysis is also conducive to meeting the demand for carbon a source for the growth of autotrophic microorganisms [10,11]. Thus, urea C can be easily incorporated into soil microbes. Yet, little is known about which active microorganisms benefit most from the supply of urea-C under different typical agricultural practices, and how microbial urea-C utilization varies across different management types.

The paddy field is a unique agricultural ecosystem, maintained under flooded conditions for most of the rice cultivation period, with a microbial biomass that is much higher than that in upland soils [12]. The abundant microbes in paddy soil play a crucial role in the transformation and storage of exogenous carbon [13]. In recent years, irrigation management in Chinese paddy fields has shifted towards water-saving practices to cope with the rapid increase in agricultural water consumption and the competitive demands from urban and industrial sectors [14,15]. This transition from flooded irrigation (anaerobic condition) to water-saving irrigation (aerobic conditions) significantly impacts microbial activity and community structure, as soil water and oxygen condition are key regulators of microbial activity [16,17]. The variation in microbial group abundance under different irrigation regimes can further alter the fate of exogenous C and nutrient transformations. Few studies have evaluated the utilization of exogenous C by microorganisms in rice–soil systems under different irrigation regimes [18,19]. For example, lower soil microbial carbon dioxide fixation rates are observed under non-flooded conditions compared to flooded conditions in paddy soils [10,20]. In contrast, more photosynthetic C is incorporated into soil microbial communities under non-flooded conditions [21,22]. Variations in C influx into microbial groups due to different agricultural practices can significantly affect the terrestrial C cycle and storage. Information on microorganisms associated with urea-C utilization in relation to water regimes in rice systems is particularly limited and urgently needed.

Furthermore, water-saving irrigation induces physiological and biochemical changes in rice plants, increasing root metabolic activity and developing more fine roots [14,23]. These changes affect the quality and quantity of photosynthesized carbon, substantially influencing the structure and function of the microbial community in the rice rhizosphere [18,21]. Rhizosphere microbial communities exert significant local control on C and nutrient cycles, suggesting plant-induced changes in soil microbial communities may play previously unappreciated roles in the soil C cycling in agricultural ecosystems [24,25,26]. Therefore, it is necessary to study the irrigation-mediated alteration of rhizosphere microbial community structure and its consequences for microbial urea-C utilization.

The combination of phospholipid fatty acids (PLFAs) and stable isotope probing (SIP) has provided an efficient method to quantify urea-derived C assimilation by microorganisms and to identify the specific microbial groups involved. This study aims to (*i*) investigate the changes in the microbial community composition in the rice rhizosphere and bulk soil under different irrigation regimes; and (*ii*) elucidate the differences in the groups of microorganisms assimilating urea-C between bulk and rhizosphere soil, and determine how these groups are affected by varying irrigation regimes.

## 2. Materials and Methods

### 2.1. Soil Sampling

Soil for the experiment was collected from the Kunshan Irrigation and Drainage Experiment Station (34°15′ N, 121°05′ E), located in a subtropical monsoon region of China with a mean annual temperature of 15.5 °C and mean annual precipitation of 1097.1 mm. Soil samples were collected from the plow layer (0–20 cm) and sieved through a 2 mm mesh to remove coarse plant residues, dead roots, and root-derived debris. The soil pH was 7.2. The contents of soil organic C and total N, P, K are 21.88, 1.08, 1.35 and 20.86 g·kg^−1^, respectively.

### 2.2. Experimental Design and Sampling 

**Figure 1:**
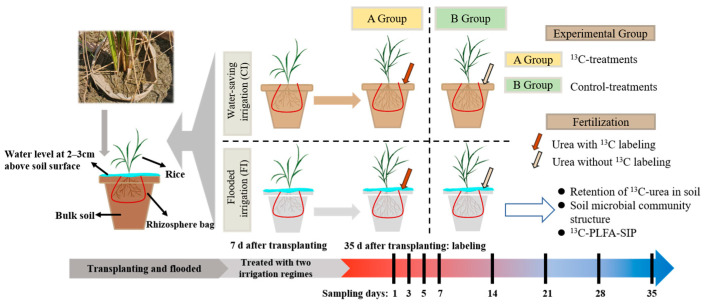
Schematic diagram of soil culture experiment.

To prevent soil from being affected by rainfall, the pot experiment was conducted under a rain shelter at the Kunshan Irrigation and Drainage Experiment Station (Figure 1). Two irrigation treatments were used in the experiment: flooded irrigation (FI) and water-saving irrigation (CI). Each treatment was replicated twenty times. In each pot, a rhizosphere bag (18 cm height × 8 cm, 30 μm mesh) was used to separate the soil into rhizosphere (within the rhizosphere bag) and bulk (out of the rhizosphere bag) soil portions. The experimental pots, with an inner diameter of 21 cm and height of 20 cm, were filled with paddy soil mixed with phosphorus and potassium fertilizers at rates of 72 and 128 mg·kg^−1^ soil, respectively. Basal nitrogen fertilizer was applied as urea without ^13^C labeling at the rate of 65 mg N·kg^−1^ soil.

Rice seedlings were transplanted into rhizosphere bags in each pot on 5 July 2020, with the water level maintained at 2–3 cm above the soil surface (three seedlings per pot). After the rice entered the regreening stage, all pots were divided into two groups for different irrigation regime treatments. Half of the pots were maintained under flooded conditions (FI) with the water levels kept 2–3 cm above the soil surface throughout the experimental period. The other half were subjected to water-saving irrigation, where the soil moisture or surface water level was close to the lower irrigation threshold and water was added manually to moisture the soil to the upper soil moisture threshold (saturated but not flooded). The soil moisture content or surface water level was maintained between the upper and the lower irrigation threshold for each specific growth stage. Detailed information on the controlled irrigation regime can be found in Xu et al. [27]. The soil moisture threshold for CI used in this study is controlled irrigation, which has been proven to be effective for saving water, reducing nitrogen runoff-loss and greenhouse gas emissions [4,27,28].

Thirty-five days after transplanting, pots from each irrigation regime were further divided into two groups: one group was the ^13^C-labeled group, to which ^13^C-labeled urea (as topdressing) was applied at the rate of 35 mg N·kg^−1^ soil. And the other group was the control group: urea without ^13^C labeling was applied at the rate of 35 mg N·kg^−1^ soil. These pots (control group) were treated in the same manner as urea-^13^C labeled pots and were placed away from the ^13^C-labeling group. The control group was sampled each time we collected samples from the ^13^C-labeled urea pots.

Rhizosphere and bulk soil samples were randomly collected from each pot 1, 3, 5, 7, 14, 21, 28 and 35d following urea application (labeling) and analyzed to determine the dynamics of urea-^13^C retention in the soil. The soil inside and outside the mesh was considered rhizosphere soil and bulk soil, respectively. Three cores were taken with a soil sampler from the bulk area (non-rhizosphere soil). These subsamples were mixed and homogenized into a composite bulk soil sample for each pot. Meanwhile, soil collected inside the rhizosphere bag was carefully removed from the roots before being pooled and homogenized to form a composite rhizosphere soil sample for each plot. As roots and soil fungi share several PLFA biomarkers; to ensure that the PLFA profiles and their δ^13^C values represent the soil microbial community rather than plant materials, any roots adhering to the soil sample that were difficult to separate were removed as described by Butler et al. [29] and Liua et al. [30]. Specifically, the rhizosphere soil samples were suspended in 40 mL of 50 nM phosphate buffer (pH 6.8) and centrifuged at 8400× *g* for 5 min. The supernatant was then decanted to remove floatable and root-derived debris. Many studies have shown that the sieving centrifugation procedure is effective [30]. All fresh rhizosphere and bulk soil samples obtained were dried at 50 °C to a constant weight and then ground and sieved through a 100 mesh. The 7 and 21d fresh rhizosphere and bulk soil samples were divided into two subsamples. One subsample was immediately immersed in liquid nitrogen and stored at −70 °C for PLFA analysis. The other subsample was dried at 50 °C to constant weight before being ground and sieved through a 100 mesh for determining δ^13^C values. Rice plants were cut off at the base, allowing for separation of the leaf and shoot, 35d after urea application (labeling). The leaf, shoot and root (including visible fine roots separated from rhizosphere soil) samples were cleaned with water, dried at 50 °C to a constant weight, weighed and then ground for analysis. 

### 2.3. Soil PLFA Extraction and Analysis

Phospholipid fatty acids (PLFAs) were used to quantify changes in microbial biomass in our experiment and to compare the label C uptake between different microbial groups. Soil PLFA, either from the rhizosphere or the bulk soil, were extracted, fractioned, and purified as described by Frostegrd et al. [31]. Briefly, freeze-dried soil samples were extracted twice using 22.8 mL of a one-phase extraction mixture containing chloroform–methanol–citrate buffer (1:2:0.8, 0.15 M, pH 4.0). Silica acid columns (Supelco, Bellefonte, PA, USA) were used to separate phospholipids from glycolipids and neutral lipids. Phospholipids were methylated using a mild alkaline methanolysis to derivatize them to their corresponding fatty acid methyl esters (FAMES) [32]. Methyl nonadecanoate fatty acid (19:0) was added as an internal standard. PLFA methyl esters were separated and identified using gas chromatography (GC 7890A; Agilent, Santa Clara, CA, USA) and the MIDI Sherlock microbial identification system (MIDI Inc., Newark, DE, USA). The PLFA numbers are listed in Table 1.

### 2.4. ^13^C in Plant, Soil and PLFA

The C concentration, atom% ^13^C and δ^13^C signature of the soil, leaf, shoot and root pools were determined using a Flash 2000HT elemental analyzer (Thermo Fisher Scientific, Waltham, MA, USA) interfaced to an isotope mass spectrometer (DeLTA V Advantage, Thermo Fisher Scientific) (EA-IRMS). The retention of urea-^13^C in the soil or plant was calculated as follows:

(1) Rretention=Ctotal×(Cat%13)Labeled−(Cat%13)ControlCaddition×100%

where *C_total_* is the total C content in the soil or plant sample, (^13^*C_at%_*)*_Labeled_* and (^13^*C_at%_*)*_Control_* are ^13^C atom% in the labeled and control samples, respectively, and *C_addition_* is the ^13^C inputs.

The ^13^C/^12^C ratios of individual PLFAs were analyzed by GC-C-IRMS using a Trace GC Ultra gas chromatograph with combustion column attached via a GC Combustion III to a Delta V Advantage isotope ratio mass spectrometer (Thermo Fisher Scientific, USA).

The δ^13^C value of each PLFA molecule was corrected for the C added during derivatization using a mass balance calculated according to Equation (3) [42].

(2) ncdδCcd13=ncδC13c+ndδC13d

where *n* is the number of C atoms, *n_c_* is the number of C atoms of underivatized compound, *n_d_* is number of C atoms of derivatizing agent (methanol, *n_d_* = 1 and δ^13^C value = −29.33‰), *n_cd_* is number of C atoms of corresponding derivatized compound [36,43,44].

The amount of urea-derived labeled C in each PLFA (*Pi*) was calculated using Equation (3):

(3) Pi=Mi×(δC13Labeled−δC13Control)/(δC13Urea−δC13Control)

where *Mi* is the molecular C content of the individual PLFAs, *δ*^13^*C_Labeled_* is the δ^13^C of the individual PLFAs in the labeled group, *δ*^13^*C_Control_* is the δ^13^C of individual PLFAs in the control group, *δ*^13^*C_Urea_* is the δ^13^C of urea.

The relative abundance of individual ^13^C-labeled PLFA (*P_r_*) in each soil was calculated according to Equation (4):

(4) $$P_{r}=Pi/\sum Pi$$

### 2.5. Statistical Analysis

The data were analyzed using SPSS 20.0 (SPSS Inc., Chicago, IL, USA). The least significant difference at the 95% level was calculated by a one-way analysis of variance (ANOVA) to determine differences between treatments on the same sampling day. The molar percent of the individual PLFA to the total PLFAs and the relative abundance of individual ^13^C-labeled PLFA were standardized to the unit variance (scaling) after generating a correlation matrix and were then subjected to principal component analysis (PCA) to explain the variations in data as described by Wang, Thornton and Yao [42] and Yao, Thornton and Paterson [22]. PCA of PLFAs was performed using an app named Principal Component Analysis (V1.50) in origin 2021b. All parameters were estimated using the Origin 2021b software (Origin Lab corporation, Northampton, MA, USA).

## 3. Results

### 3.1. ^13^C-Urea Remained in Soil

**Figure 2:**
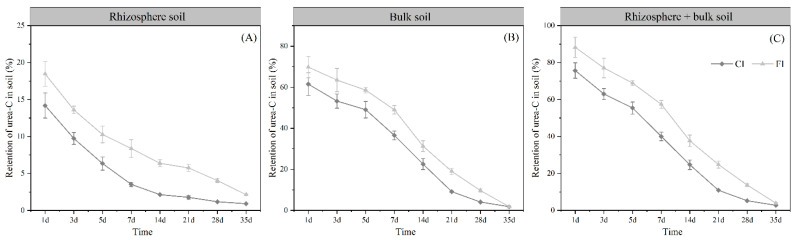
Retention of urea-^13^C in rhizosphere soil (**A**), bulk soil (**B**) and rhizosphere + bulk soil (**C**).

After 1 day, a total of 75.70–88.27% of the urea-C remained in the rhizosphere and bulk soil, suggesting that the hydrolysis of urea may be immediate in soils under different irrigation regimes and that a portion of the urea-C had been lost. Then, the residual urea-C in both the rhizosphere and bulk soil decreased rapidly over time. The urea-^13^C remaining in the rhizosphere and bulk soil under both irrigation regimes generally followed the same pattern, with the values in the rhizosphere soil being consistently higher than those in the bulk soil, and the values in FI being higher than in CI (Figure 2). Thirty-five days after urea-^13^C application, there were no significant differences between the urea-^13^C retained in the rhizosphere soil and bulk soil, with only a small amount (less than 2%) of urea-^13^C remaining in the soil.

### 3.2. Soil Microbial Community Structure

**Figure 3:**
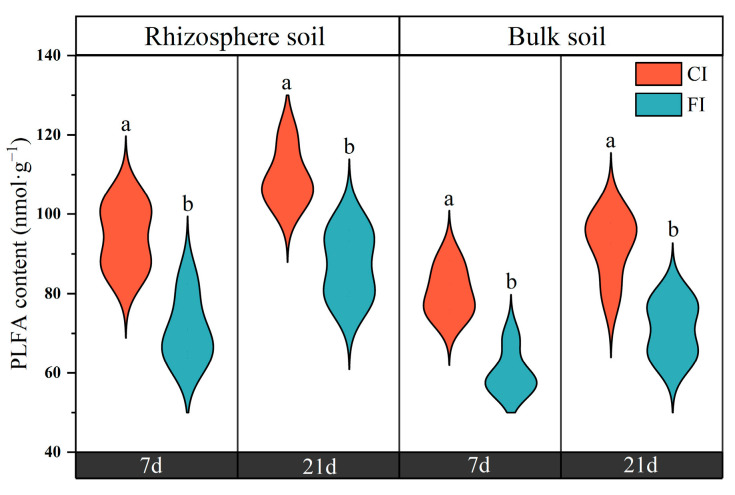
Total amounts of PLFAs on the 7th and 21st days. Different letters above the bars indicate significant differences at *p* < 0.05 between CI and FI treatment.

**Figure 4:**
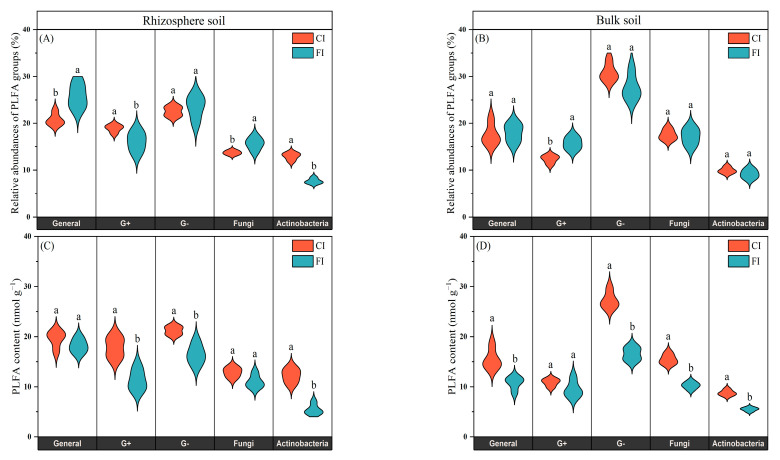
Composition (**A**,**B**) and content (**C**,**D**) of PLFAs under different treatments. Different letters above the bars indicate significant differences at *p* < 0.05 between CI and FI treatment.

The total amount of PLFAs on the 21st day was slightly higher than that on 7th day in the different soil samples (Figure 3). The CI treatment significantly increased the total amount of PLFA in both the rhizosphere and bulk soil compared with the FI treatment, with the total amount of PLFAs being higher in rhizosphere soil than in bulk soil. Under CI treatment, the contents of Gram-negative bacteria (G−), General bacteria, Fungi and Actinobacteria in the bulk soil and Gram-positive bacteria (G+), G− and Actinobacteria in the rhizosphere soil were significantly higher than those under FI treatment (*p* < 0.05, Figure 4C,D).

**Figure 5:**
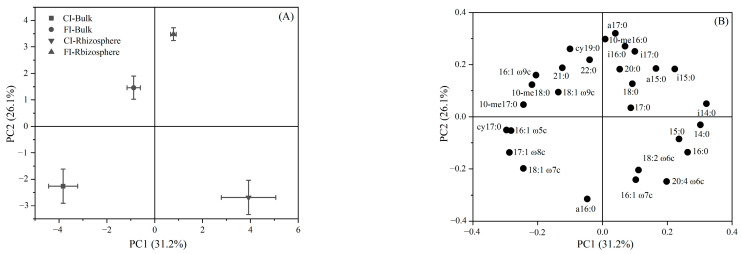
(**A**) Principal component analysis (PCA) based on the relative abundance of individual PLFA in soil samples at seventh day and (**B**) a loading plot of two components from the PCA on the PLFA component. Values in parentheses on the axis labels indicates the percentage variation accounted for by each axis.

The proportions of microbial groups in the microbial community were calculated for all treatments on the 7th and 21st days after C labeling. The relative abundances of the six microbial groups remained relatively constant and did not differ between the 7th and 21st days after urea application (Figure 4 and Figure S1). For clarity, only data for the 7th day are presented in Figure 4 and Figure 5. Although the CI treatment increased the content of PLFA groups in bulk soil, the soil microbial community composition in the bulk soil was similar under both CI and FI treatments. The relative abundances of the G−, General bacteria, Fungi, Actinobacteria remained fairly constant, except for a significant increase in G+ with the FI treatment (Figure 4B). In contrast, the irrigation regime had more significant effects on the microbial community structure in the rhizosphere soil. Compared to the FI treatment, the CI treatment significantly increased the relative abundances of the G+ and Actinobacteria in the rhizosphere soil (*p* < 0.05, Figure 4A). We also observed that FI treatment significantly increased the relative abundances of the General bacteria and Fungi in the rhizosphere soil compared to the CI treatment (*p* < 0.05). There was no difference in the relative abundances of G− between CI and FI treatments in the rhizosphere soil. 

A principle component analysis (PCA) on the relative abundances of PLFA was used to assess the differences in the microbial community composition among the different treated soil samples. All treatments were clearly distinguished from each other, indicating that irrigation regime changed the active microbial community composition in both the rhizosphere and bulk paddy soils (Figure 5A). Factor loadings of PLFAs suggested that a16:0, 16:1ω7c and 20:4ω6c were relatively enriched under CI treatment, while a17:0, 10-me16:0 and i16:0 were relatively enriched under FI treatment. The loadings also confirmed that the PLFAs 16:1ω5c, cy17:0 and17:1ω8c were relatively enriched in bulk soil, while 14:0, i14:0 and 16:0 were relatively enriched in the rhizosphere soil under the different irrigation regimes (Figure 5B).

### 3.3. ^13^C-PLFA-SIP

**Figure 6:**
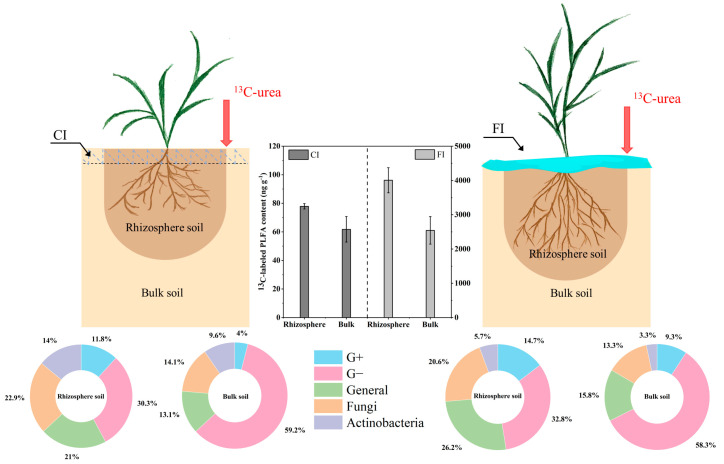
Total ^13^C-labeled PLFA content and the relative abundance ^13^C-labeled PLFAs in the different microbial groups in soil sampled 7 days after urea application.

**Figure 7:**
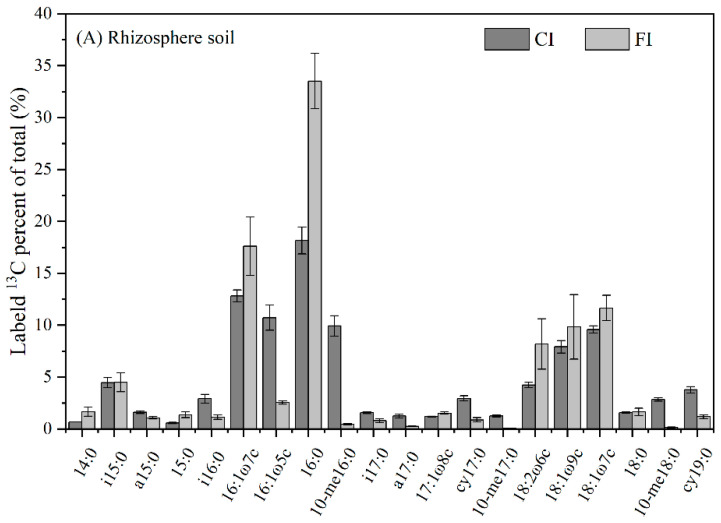

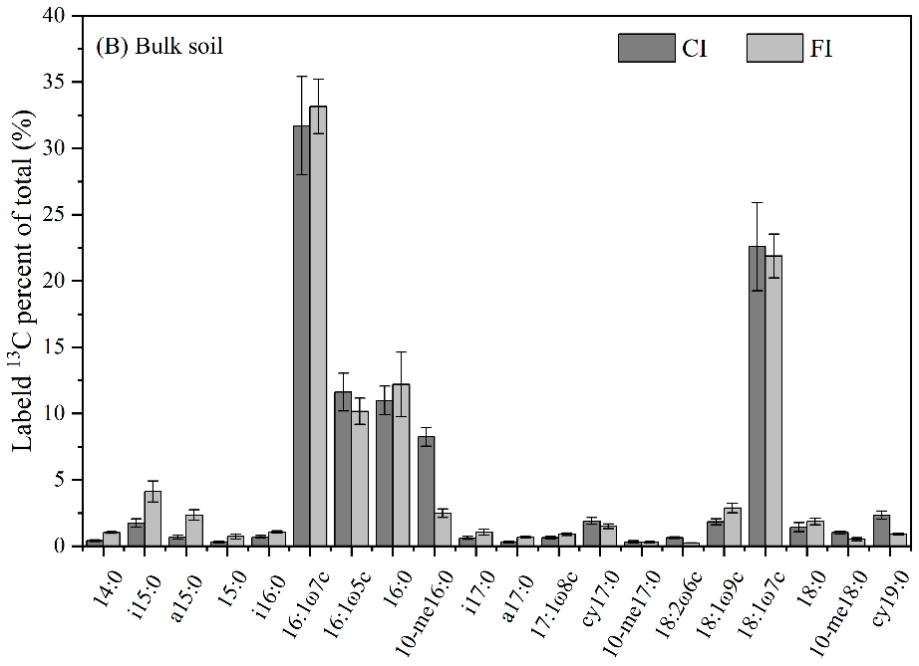
Relative abundance of individual ^13^C-PLFA from urea-derived ^13^C in soil sampled 7 days after urea application.

**Figure 8:**
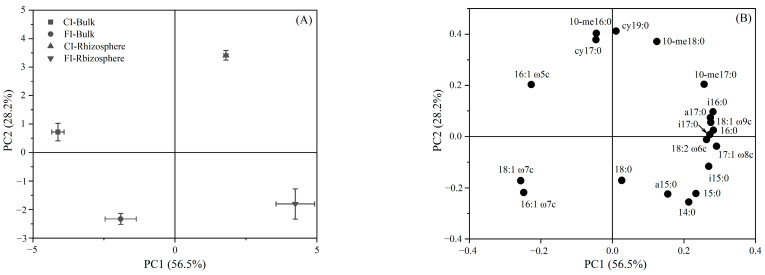
(**A**) Principal component analysis (PCA) of ^13^C-PLFA composition in soil samples on the seventh day and (**B**) a loading plot of the two the PCA on the PLFA components. Values in parentheses on the axis labels indicate the percentage variation accounted for by each axis.

The pattern of urea-^13^C incorporation into PLFAs did not differ between 7 and 21 days after urea application (Figure S2); for clarity, we only present data for day 7 (Figure 6, Figure 7 and Figure 8). The ^13^C-labeled PLFA content under the FI treatment was much higher than that under the CI treatment in both rhizosphere and bulk soil, with more labeled C derived from ^13^C-urea found in the PLFAs in the rhizosphere soil. We further calculated the relative ^13^C-labeled PLFA contents of the different microbial groups. The relative ^13^C distribution showed that most ^13^C-labeled PLFAs derived from the urea were primarily G− followed by General bacteria under both rhizosphere and bulk soil. The ^13^C originating from the urea was not evenly distributed among the soil microbial groups, indicating that the soil microorganisms differed in their uptake and utilization of ^13^C-urea. The relative abundance ^13^C-labeled PLFA of the rhizosphere and bulk soil microbial groups under the CI and FI treatments were consistent and followed the order G− and General bacteria > Fungi > G+ and Actinobacteria (Figure 6). Despite these general trends, the irrigation regimes had different effects on the relative ^13^C-labeled PLFA contents of the different microbial groups in the rhizosphere and bulk soil. Compared with the FI treatment, the CI treatment resulted in a higher percentage of ^13^C-labeled PLFAs in the G−, Fungi and Actinobacteria, but a lower percentage in the G+ and General bacteria in the bulk soil. Similarly, in the rhizosphere soil, the CI treatment resulted in a higher ^13^C-labeled percentage of Fungi and Actinobacteria, but a lower percentage of G+, General bacteria and G− (Figure 6).

Twenty different PLFAs were involved in ^13^C-urea. Most labeled urea-C was incorporated into 16:1ω7c, 16:0 and 18:1ω7c. The PLFAs 16:0, 16:1ω5c, 16:1ω7c, 10-me16:0, 18:1ω7c and 18:1ω9c accounted for 66.62–86.98% of the labeled phospholipids derived from ^13^C-urea under the different treatments (Figure 7). Despite these general trends, the pattern of ^13^C incorporation into the PLFA pool differed between the different treatments.

The relative abundances of ^13^C-PLFAs under different treatments were separated according to the principal component analysis. The bulk and rhizosphere soil under the CI and FI treatments were separated along PC1, accounting for 56.5% of the variation (Figure 8A). The factor loadings of individual PLFAs suggested that PLFAs 18:1ω7c, 16:1ω7c and 16:1ω5c were relatively enriched in urea-C in the bulk soil, while 17:1ω8c, i16:0, 16:0 and 18:1ω9c were relatively enriched in urea-C in the rhizosphere soil under the different irrigation regimes. The loadings also confirmed that 10-me16:0, cy17:0 and cy19:0 were relatively enriched in urea-C under the CI treatment, while 14:0, a15:0 and 15:0 were relatively enriched in urea-C under the FI treatment (Figure 8B).

## 4. Discussion

### 4.1. The Retention of Urea-C

**Figure 9:**
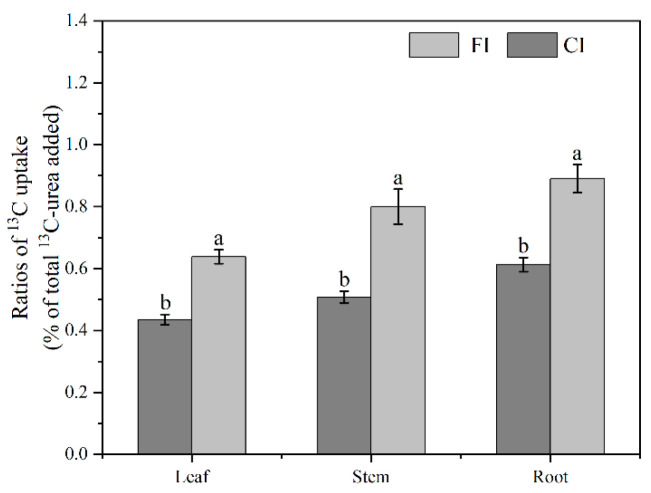
Urea-C uptake by rice. Different letters above the bars indicate significant differences at *p* < 0.05 between CI and FI treatment.

The dynamics of urea-^13^C retention in the soils followed different patterns between the different irrigation regimes. The CI treatment induced a lower retention of urea-^13^C in both the rhizosphere and bulk soil compared to the FI treatment, as the aerobic conditions under the CI treatment enhanced urease activity, favoring urea enzymatic hydrolysis [45,46]. Regardless of the irrigation regime, we also observed that the retention of urea-C in both the rhizosphere and bulk soil decreased rapidly over time, especially in the rhizosphere soil where the decrease was more pronounced. The possible reasons for this observation are (*i*) that the input of labile organics by rice roots stimulates microbial activity and therefore facilitates the rates of biochemical process in the rhizosphere compared to the bulk soil. This forms microbial hotspots (i.e., higher microbial and enzyme activity) and rhizosphere priming, resulting in more urea-C being lost in the form of CO_2_; and (*ii*) we observed that ^13^C was enriched in the rice organs under the different treatments (35 d after ^13^C-urea labeling), consistently following the order root > stem > leaf (Figure 9). This is consistent with the existing theory that plants possess dedicated urea transporters and can utilize urea directly (without prior soil conversion) in agricultural systems [47]. In addition, HCO_3_^−^ produced by the enzymatic hydrolysis of urea can be utilized by plant roots as a carbon source [48]. These findings might explain the differences in urea-^13^C retention between the rhizosphere and bulk soil.

### 4.2. Effect of Irrigation Regimes on Bulk and Rhizosphere Soil Microbial Community

In the current study, the effects of irrigation regimes on the microbial communities in bulk and rhizosphere soils were evaluated. As expected, various factors (such as soil moisture, oxygen concentration and nutrient availability) influenced by the irrigation regimes led to variations in microbial abundance and composition in both the bulk and rhizosphere soils (Figure 3, Figure 4 and Figure 5). CI treatment significantly increased the total amount of PLFAs in both the rhizosphere and bulk soils compared to the FI treatment. This increase may be attributed to the CI treatment providing oxygen and creating an appropriate soil moisture status for microorganisms, potentially inducing indirect effects by altering nutrient availability, redox conditions, pH and soil aggregation. These factors collectively create more suitable habitats and microenvironments for microorganisms, thereby benefiting microbial communities [14,49,50]. This finding is consistent with previous studies indicating that the change from oxygen-limited conditions (flooded irrigation) to aerobic conditions (water-saving irrigation) enhances microbial growth and activity in paddy soils [46,51].

The irrigation regime exerted differential effects on the soil microbial community in bulk and rhizosphere soil. Although CI treatment increased microbial biomass, it only significantly reduced the abundance of G+, with no significant effects being observed on the relative abundances of other microbial communities in the bulk soil compared to the FI treatment (Figure 4). It can be inferred that bulk soil microbial biomass may be more sensitive to soil moisture variations than microbial community composition. In contrast, irrigation regime-induced shifts in microbial community structure were more pronounced in the rhizosphere soil than in the bulk soil. Distinct categories and the availability of carbon substrates are the most common limiting factors affecting microbial community structure and activity in rhizosphere soil [52]. Root-deposited photosynthate represents a critical source of readily available carbon for microorganisms in the rhizosphere soil [53]. Variations in the quantity and composition of rhizodeposits from rice roots during rice growth likely contribute to the differences in the rhizosphere soil microbial communities between the different irrigation regimes observed in this study. Previous studies have shown that root exudates respond rapidly to water regimes, with higher root activity observed under non-flooded and alternating wetting and drying conditions compared to continuously flooded conditions, potentially leading to increased rhizodeposition [18,19,21]. The organic substances within root-exudates may lead to a re-structuring of the composition of rhizosphere soil microbial communities, especially fast-growing communities with the ability to utilize various root-derived carbon substrates [54]. 

### 4.3. Incorporation of Urea-C into Bulk and Rhizosphere Soil Microbial Groups under Different Irrigation Regimes

An important objective in previous PLFA ^13^C labeling experiments was to determine the utilization of exogenous carbon by soil microorganisms. The results of this study demonstrated that, although the amount of PLFA in soil undergoing water-saving irrigation was significantly higher than that undergoing flooded irrigation, the incorporation of urea-^13^C into both rhizosphere and bulk soil PLFAs was much lower in under the CI compared to the FI treatment (Figure 6). These results suggest that soil microbes in flooded paddy field have a higher capacity for assimilating intact urea molecules and their hydrolyzed products, and more urea-derived C may be recovered in the microbial necromass pools, consequently contributing to the SOC sequestration [55].

Total ^13^C-lableled PLFA abundance revealed that G− (especially 16:1ω7c and 18:1ω7c) and General bacteria (especially 16:0) actively utilize more urea-C, making these microorganisms important urea-C sinks under both FI and CI conditions in paddy soils (Figure 6 and Figure 7). It is notable that G− and fungi have also been observed to have a high utilization rate of plant- and biochar derived-C in recent studies [19,36,56]. In line with these findings, our observation suggests that G− bacteria are competitive not only for easily available organic C from straw or biochar, but also for intact urea molecules and their hydrolyzed inorganic C.

Despite the different effects of irrigation regimes on the relative contents of ^13^C-labeled PLFAs in various microbial groups in the rhizosphere and bulk soils, a higher percentage of ^13^C-labeled PLFAs in Fungi and Actinobacteria was observed in both the bulk and rhizosphere soils under the CI treatment compared with the FI treatment (Figure 6 and Figure 8). This indicates that these microorganisms are more active in processing urea-C and show higher contributions to its utilization in water-saving paddy soils. This can be explained by the fact that Fungi and Actinobacteria maintain a high metabolic activity during drying periods. This capability is attributed to their ability to accumulate osmoregulatory solutes that do not impair metabolism and to their filamentous structure, which allows them to reach and exploit exogenous carbon during drying periods by bridging the air-filled pores with low diffusivity hyphae [57,58]. In contrast, the FI treatment resulted in a higher percentage of ^13^C-labeled PLFAs in G+ in both the bulk and rhizosphere soils compared with the CI treatment (Figure 6). Similarly, Tian et al. [21] reported that G+ are more active in processing rhizodeposition-C in continuously flooded paddy soils, suggesting that G+ play a prominent role in the utilization of exogenous carbon under flooded conditions. The observed differences in the utilization of exogenous carbon by microbial communities under different irrigation regimes highlight the varying underlying mechanisms of microbial-mediated carbon sequestration between non-flooded and flooded soils [59]. Fungi and Actinobacteria were more active in processing urea-derived carbon (urea-C) during drying periods in water-saving paddy soils, while G+ bacteria played a more prominent role in carbon processing under flooded conditions. These differences highlight the need to consider microbial composition and activity when designing irrigation strategies for optimal carbon sequestration and soil health. Further work is needed to validate these findings under field conditions and to conduct more intensive investigations into the effects of rice cultivation on soil microbial communities. This will allow for a deeper understanding of soil microbial structure, carbon metabolism preferences, functions and how they are influenced by crop cultivation and soil microenvironments.

## 5. Conclusions

In conclusion, water-saving irrigation treatment significantly increased the total amount of PLFA in both bulk and rhizosphere soils compared to flooded irrigation treatment, with rhizosphere soil showing a higher total amount of PLFA than bulk soil. In addition, the bulk soil microbial biomass was more vulnerable to irrigation regimes than microbial community composition. In contrast, shifts in microbial community structure induced by irrigation regimes were more pronounced in the rhizosphere soil than in the bulk soil. Stable isotope probing of the PLFAs provided greater insight than simple quantifications. G− (especially 16:1ω7c and 18:1ω7c) actively utilized more urea-C, making these microorganisms important urea-C sinks in paddy soils. Additionally, water management influenced the utilization of urea-C by microorganisms in both the rhizosphere and bulk soils. Specifically, G+, Fungi and Actinobacteria played prominent roles in the utilization of urea-C under water-saving and flooded paddy soils, respectively. The patterns of urea-C utilization by rhizosphere and bulk soil microorganisms varied with different water management practices. Here, we highlight the significant role of water management in determining microbial biomass, community structure and function in both bulk and rhizosphere soils. Further research is needed to confirm these findings under field conditions and to develop effective measures that enhance the deposition of urea-derived C into the SOM reservoir via microbial uptake, biosynthesis, growth, death and necro mass accumulation.

## Figures and Tables

**Table 1:** Phospholipid fatty acid (PLFA) biomarkers identified in the sampled soil.

Functional Group and Fatty Acid Markers		
General	14:0, 15:0, 16:0, 17:0, 18:0, 20:0, 21:0, 22:0	[33]
Gram-positive bacteria (G+)	i14:0, i15:0, a15:0, i16:0, a16:0, i17:0, a17:0	[34,35]
Gram-negative bacteria (G−)	16:1ω7c, 16:1ω9c, cy17:0, 17:1ω8c, 18:1ω7c, cy19:0	[34,36,37]
Actinobacteria	10-me16:0, 10-me17:0, 10-me18:0	[36,38]
Fungi	16:1ω5c, 18:1ω9c, 18:2ω6,9c	[39,40,41]

## Data Availability

Data supporting the findings of this study are available on request from the corresponding author.

## Supplementary Materials

The following supporting information can be downloaded at: https://www.mdpi.com/article/10.3390/microorganisms12091829/s1, Figure S1: Relative abundances of PLFA groups under different treatments on the 21st days after C labelling; Figure S2: Relative abundance of individual ^13^C-PLFA from urea-derived ^13^C in soil sampled 21st days after C labelling.
