# Supplementary material for: Water Management-Mediated Changes in the Rhizosphere and Bulk Soil Microbial Communities Alter Their Utilization of Urea-Derived Carbon
Source: Microorganisms. 2024 Sep 4;12(9):1829. doi: 10.3390/microorganisms12091829 (PMC11434454; doi:10.3390/microorganisms12091829)
Supplement: Supplementary file 1 [file microorganisms-12-01829-s001.zip › microorganisms-3132404-supplementary.pdf]

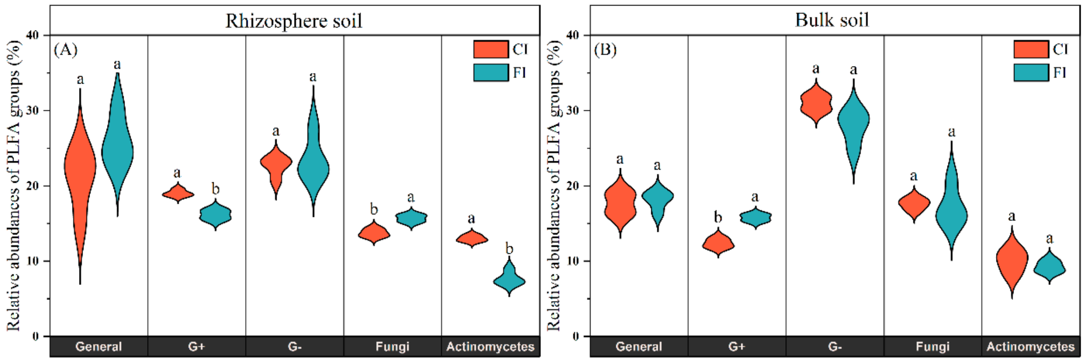

Figure S1 Relative abundances of PLFA groups under different treatments on the 21st days after C labelling. Different letters above the bars indicate significant differences at $p < 0.05$ between CI and FI treatment.

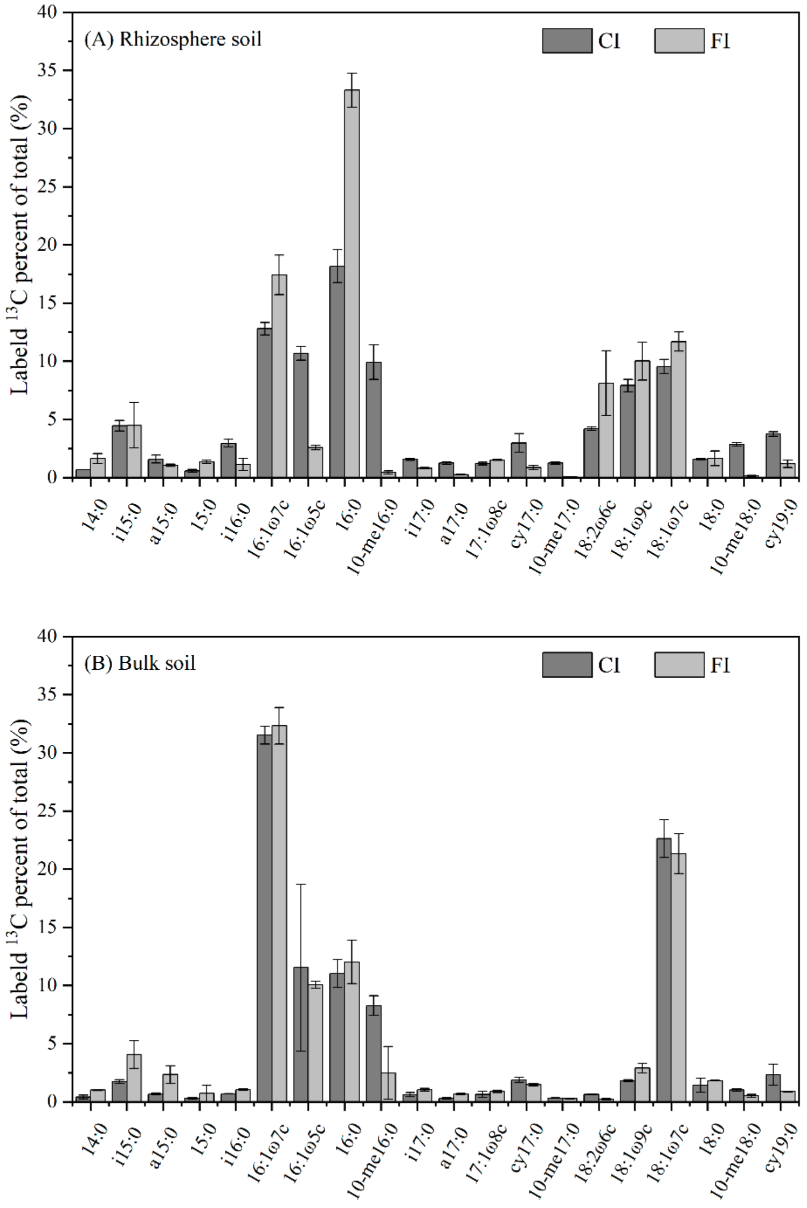

Figure S2 Relative abundance of individual ^{13}C-PLFA from urea-derived ^{13}C in soil sampled 21st days after C

labelling.
